# Hyponatremia and extrapontine myelinolysis in a patient with COVID‐19: A case report

**DOI:** 10.1002/ccr3.4463

**Published:** 2021-07-09

**Authors:** Muhammad Abd Ur Rehman, Abdulrahman Fadhl Abdulrahman, Aariz Zainab, Yahya Paksoy, Nadir Kharma

**Affiliations:** ^1^ Emergency Medicine Hamad Medical Corporation Doha Qatar; ^2^ Internal Medicine Hamad Medical Corporation Doha Qatar; ^3^ Radiology Department Hamad Medical Corporation Doha Qatar; ^4^ Critical Care Medicine Hamad Medical Corporation Doha Qatar

**Keywords:** COVID‐19, extrapontine myelinolysis, hyponatremia, ODS

## Abstract

Until we have strong evidence to the contrary, symptomatic hyponatremia should be treated with extra caution in COVID‐19 co‐infection patients as the latter could be another risk factor for the development of extrapontine myelinolysis.

## Graphical abstract

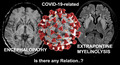

## 1 INTRODUCTION

We present this case that led us to suspect an association between COVID‐19 and extrapontine myelinolysis (EPM). It is, to the best of our knowledge, the first reported case of extrapontine myelinolysis in a patient with COVID‐19 infection. We found intertwined findings of COVID‐19‐related encephalopathy and EPM in MRI of the patient who showed significant improvement with low‐dose levodopa.

Since it was first reported in 1959,^1^ hundreds of case reports have been published on osmotic demyelination syndrome worldwide. It was Patricia J. et al. who raised the suspicion of association of hyponatremia with central pontine myelinolysis in 1977^2^ and finally, four years later, rapid correction of hyponatremia was found to be the cause of central pontine myelinolysis (CPM) in rats.^3^

Isolated extrapontine myelinolysis (EPM), a form of osmotic demyelination, is a rare yet the most feared complication of hyponatremia correction treatment that can develop due to rapid correction of hyponatremia. A few case reports suggest successful use of high‐dose levodopa for the treatment of EPM in patients who present with Parkinson's‐like symptoms.^4^

After the declaration of COVID‐19 as a pandemic by the World Health Organization (WHO), it emerged as a serious public health challenge. We are reporting a thought‐provoking case of a patient with COVID‐19 infection with suspected iatrogenic development of osmotic demyelination syndrome whose brain imaging was suggestive of an intertwined picture of COVID‐19‐related encephalopathy along with isolated extrapontine myelinolysis. Moreover, the patient showed significant improvement after using low‐dose levodopa.

## 2 CASE DESCRIPTION

A 43‐year‐old man with a known history of type 2 diabetes and essential hypertension, controlled with oral hypoglycemics and combined antihypertensive drugs, respectively, presented to one of the designated COVID‐19 facilities in our country with complaints of fever and cough for two days. Initial investigations revealed that he had mildly symptomatic COVID‐19 pneumonia diagnosed by testing a nasopharyngeal swab sample using polymerase chain reaction (PCR) and chest X‐ray. So, he was admitted for treatment and isolation.

In his initial investigations, he had significant hyponatremia (123 mmol/L) with raised serum creatinine (143 µmol/L) level while urea, potassium, and liver function tests (LFT) were within normal limits (Table 1). Management was initiated with a standard COVID‐19 treatment regimen including azithromycin, amoxicillin/clavulanate, hydroxychloroquine (HCQ), and lopinavir/ritonavir in appropriate doses.

**TABLE 1:** Important blood investigations

	At presentation (Day 0)	At the time of Hyponatremia Symptoms (Day 9)	At the time of Resolution of Symptoms (Day 12)	At the time of Osmotic Demyelination Symptoms (Day 16)
Sodium (mmol/L)	123	93 and 88^a^	126	127
Potassium (mmol/L)	3.7	3.5	3.9	3.6
Chloride (mmol/L)	84	52.7	93.4	89.1
Calcium (mmol/L)	2.07	‐	2.24	2.27
Hemoglobin (gm/dL)	14.6	15.4	13.9	12.0
WBCs (×10^3^/µL)	5.3	16.73	14.72	12.6
Platelets (×10^3^/µL)	130	257	295	267
Urea (mmol/L)	6.6	5.3	6.0	8.1
Creatinine (µmol/L)	143	72	104	82
Alanine Transaminase (U/L)	32	403	271	145
Aspartate Transaminase (U/L)	31	169	63	40
Alkaline Phosphatase (U/L)	47	67	56	‐

^a^ sample repeated after 4 hours.

He remained asymptomatic for more than a week and serum electrolytes were never repeated till the ninth day of admission when he became disoriented and started having incomprehensible speech and an unsteady gait. At this point, a computed tomography (CT) scan of the head along with blood investigations, including serum electrolytes, was urgently obtained. The CT scan was reported as normal, but the patient's serum sodium level was critically low (93 mmol/L) which fell to an extremely low value of 88 mmol/L in the next four hours (Table 1). Serum potassium and renal function tests (RFT) were within normal limits with deranged LFTs, most likely related to medications.

The patient was shifted to the intensive care unit (ICU) for correction of hyponatremia and neurologic observation. His initial Glasgow Coma Scale (GCS) score was 14/15, with some disorganized speech and an unsteady gate. Power, reflexes, and muscle tone were within normal limits. He received hypertonic saline (3% normal saline) for the next two days, resulting in a sodium level of 126 mmol/L (on the third day) which subsequently improved his condition clinically by day four. He was fully oriented to time, place, and person with explicit language. His GCS dropped to 10/15 on the fifth day, and he became less responsive with disorientation to time. On examination, he was moving all four limbs with osteotendinous reflexes of 3/4 in all the extremities. A repeat CT head scan revealed no ischemic changes. This was followed by a magnetic resonance imaging (MRI) of the brain which was also reported as unremarkable.

**FIGURE 1:**
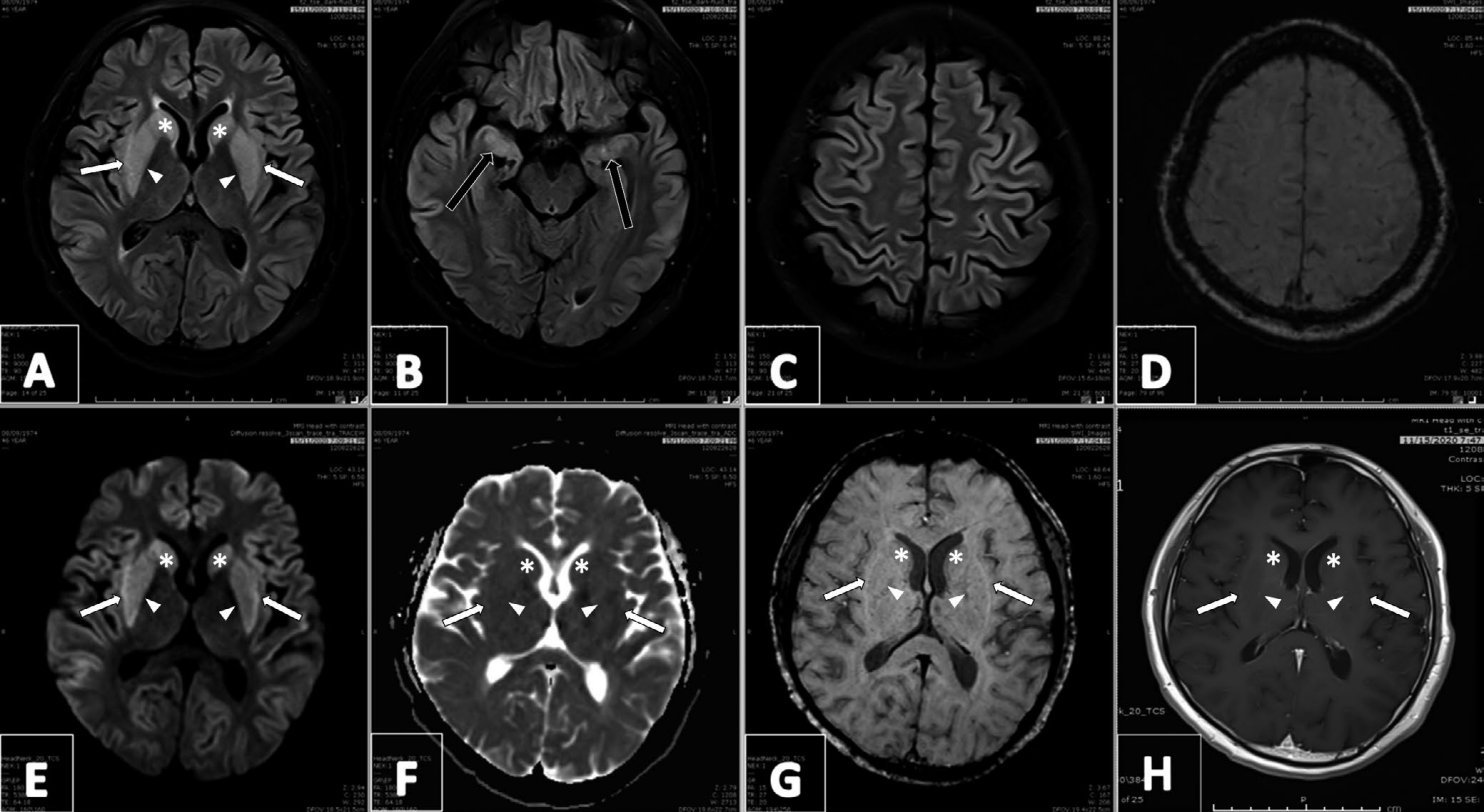
Magnetic Resonance Imaging (MRI) of the brain. High signal intensity on T2 FLAIR (A) and DWI (E), while hypointense signal on T1‐weighted images (H) along with corresponding low signal on ADC map (F) signify edema and restricted diffusion due to osmotic demyelination. Notice bilateral amygdala hyperintensity (black arrow in B) along with cortical gray matter swelling (C), showing COVID‐19‐related encephalopathic changes. No SWI changes (D, G). Symbols point out the involved regions, that is, caudate nuclei (asterisk), putamen (arrowhead), and external capsule (white arrow)

On day seven, his GCS further declined to 8/15 and he was intubated and transferred to a tertiary care facility for further management. The MRI was repeated after almost two weeks from the start of the symptoms and is shown in Figure 1. T1‐weighted images (T1WI) revealed hypointense signals this time, whereas T2‐weighted images (T2WI), T2‐weighted fluid‐attenuated inversion recovery (T2 FLAIR), and diffusion‐weighted images (DWI) revealed high signal intensity in the caudate nuclei, putamen, and external capsule bilaterally, with a corresponding low signal on the apparent diffusion coefficient (ADC) map, indicating edema and restricted diffusion due to osmotic demyelination. Of particular note in this MRI study were the findings of bilateral amygdala hyperintensity on T2WI and FLAIR sequence shown in Figure 1B. This, with bilateral cortical gray matter swelling, goes with COVID‐19‐related encephalopathic changes. Over the next two days, the patient developed an increase in muscle tone, especially in the upper limbs. A low dose of carbidopa/levodopa (12.5/50 mg thrice daily) was started to ameliorate muscle rigidity. Five days later, he was successfully extubated and shifted to the medical floor.

**FIGURE 2:**
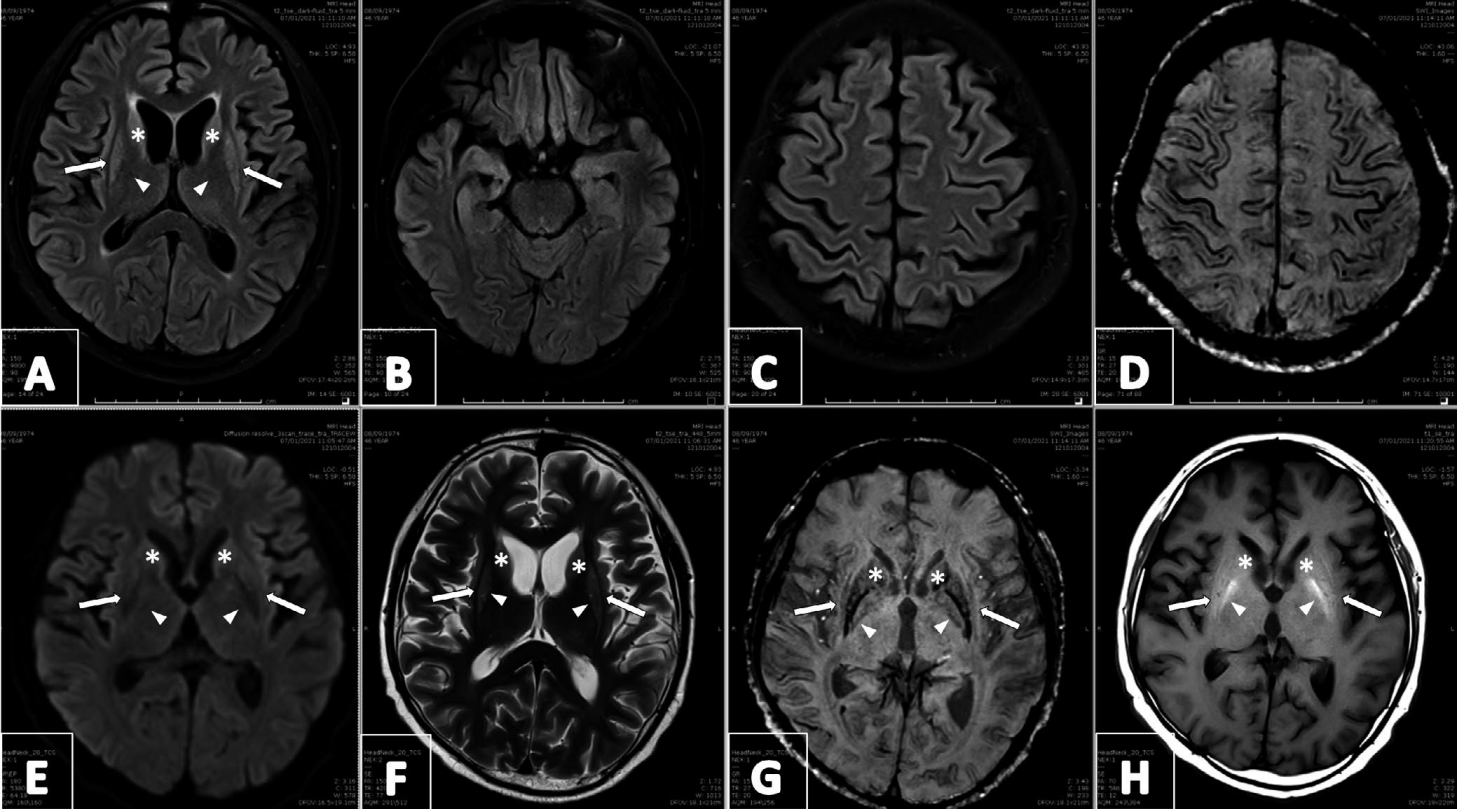
Magnetic Resonance Imaging (MRI) of the brain. (Follow‐up). Significant decrease of symmetrical swelling and abnormal signal intensity in the involved regions as compared to earlier MRI (A–F, H). SWI sequence (G) displays susceptibility changes in the parietal, subcortical, putamen, and caudate regions, suggestive of microhemorrhages due to COVID‐19‐related encephalopathy. Symbols are point out the involved regions, that is, caudate nuclei (asterisk), putamen (arrowhead), and external capsule (white arrow)

A follow‐up MRI performed nearly two months after the onset of symptoms revealed a significant interval decrease in the previously described symmetrical swelling and abnormal signal intensity in the bilateral caudate nuclei, putamen, external capsule, and cortical gray matter (Figure 2). There was a signifying improvement in edema of osmotic demyelination. The susceptibility‐weighted imaging (SWI) sequence, which was unremarkable in previous Imaging, displayed susceptibility changes in the parietal, subcortical, putamen, and caudate regions in this MRI, suggestive of microhemorrhages due to COVID‐19‐related encephalopathy (Figure 2G).

After four months of follow‐up, the patient showed considerable improvement in his cognitive and functional status. At the time of reporting this case, he was alert and oriented, but still needed assistance for physical activity.

## 3 DISCUSSION

Osmotic demyelination syndrome refers to a phenomenon in which myelin from neural cells, especially oligodendrocytes, undergoes apoptosis due to the abrupt movement of intracellular water from the brain cells into the extracellular compartment. This condition has different etiologies and one of those is rapid correction of hyponatremia, which is very obvious in our case.

Serum sodium is responsible for serum tonicity.^5^ Hyponatremia results in the drop of serum tonicity that facilitate the movement of water from the extracellular compartment into brain cells, which may result in cerebral edema if it occurs acutely and dramatically. However, the brain cells can adapt by reducing the intracellular tonicity through the transport of intracellular solutes to the extracellular compartment. Through this mechanism, the brain cells remove the excess water inside the cells to prevent cerebral edema. This adaptation process usually takes 2–3 days to ensue. After these days, rapid correction of serum sodium will result in an abrupt increase in serum tonicity. Brain cells will not have the time to adapt to the new equilibrium. So, water will move extracellularly, leading to the shrinkage and apoptosis of brain cells.^5^, ^6^ This phenomenon is known as osmotic demyelination syndrome (ODS).

ODS includes two conditions, central pontine myelinolysis (CPM) and extrapontine myelinolysis (EPM). While CPM primarily affects the basis pontis, EPM mainly affects the basal ganglia, thalami, and subcortical white matter.^7^ Isolated EPM cases are rarely reported in the literature. In a large Swedish study of 83 cases of ODM, around 4% of the cases had isolated EPM.^8^ As explained before, ODS basically occurs due to the rapid correction of severe chronic hyponatremia (serum sodium less than 120 mmol/L for more than 48 h). However, there are other risk factors that increase the risk of ODS even with higher serum sodium levels. These include, but not limited to, chronic alcoholism, malnutrition, liver diseases, and hypokalemia.^9^ In chronic hyponatremia, the maximum rate of sodium correction should not exceed 8 mmol/L over 24 h.^10^ Typically, signs of ODS take 2–6 days to evolve.^11^ EPM can manifest in diverse presentations such as spastic paraparesis, mutism, catatonia, myoclonic jerks, and parkinsonian features with choreoathetosis or dystonia.^12^

Magnetic resonance imaging (MRI) of the brain remains the gold standard modality to diagnose ODM. Nevertheless, MRI may not demonstrate the radiological features of ODM up to four weeks after the clinical manifestations have been established.^13^ In our case, early MRI brain was unremarkable. Repeating brain MRI after 1–2 weeks is recommended if there is a high clinical suspicion of ODS.^14^ For our patient, repeated brain MRI (after one week) clearly showed the features of EPM. In this case, the improvement of hyperintense signal in caudate, putamen, amygdala, and cortical gray matter with the presence of micro‐hemorrhages on SWI on the follow‐up MRI was suggestive of COVID‐19‐related encephalopathy^15^ findings entwined with those of extrapontine myelinolysis which might have worsened the underlying osmotic demyelination.

Regarding the treatment for ODS, supportive therapy is essential for all ODS cases.^16^ In the early stage, relowering of serum sodium showed some benefit, but the evidence is limited to animal studies and a few case reports.^17^, ^18^ Plasmapheresis may improve the neurologic outcomes of ODS, according to some reports.^19^ Other treatment modalities, like corticosteroid and intravenous immunoglobulin, may be useful.^14^ However, all these options need to be further studied to clarify their role in ODS management. Some patients with predominant dystonia and muscle rigidity showed a considerable response to levodopa.^4^ Our patient received a low dose of carbidopa/levodopa. We appreciated an improvement in his clinical condition over more than four months of follow‐up. Furthermore, repeated MRI brain revealed a significant improvement.

## 4 CONCLUSION

COVID‐19 is undoubtedly the most studied disease in recent years, yet there is still a lot to learn about the disease pattern. This novel case with clinical and MR findings of both COVID‐19‐related encephalopathy and ODS raises a question about COVID‐19 as a risk factor for the development or worsening of ODS. Further studies are suggested to establish an association.

Moreover, previous data suggest some effectiveness of carbidopa/levodopa treatment at high doses only (at least 25/100 mg thrice daily) but we noticed marked improvement even with 50% of the dose reported in the literature. Investigating the optimal therapeutic dose of carbidopa/levodopa should be a key objective in future studies.

## CONFLICT OF INTEREST

In compliance with the ICMJE uniform disclosure form, all authors declared no conflict of interest.

## AUTHOR CONTRIBUTIONS

Muhammad Abd Ur Rehman: involved in data curation and wrote original draft. Abdulrahman Fadhl Al‐Mashdali: validated, wrote, reviewed, and edited the manuscript. Aariz Zainab: wrote, reviewed, and edited the manuscript. Yahya Paksoy: conceptualized the study. Nadir Kharma: supervised the study.

## ETHICAL APPROVAL

This article has been submitted to the Medical Research Centre in Qatar, and an approval letter was obtained from ethical committee as well as institutional review board (IRB) with approval number of MRC‐04–21–226. Copies of the above‐mentioned document are available to be presented to the editor‐in‐chief if required.

## CONSENT STATEMENT

Informed verbal consent was taken from the patient while written consent was taken from the patient's wife as the patient was unable to sign due to upper limb weakness.

## Data Availability

Data sharing is not applicable to this article as no datasets were generated or analyzed during the current study.
